# Out of Refugia: Population Genetic Structure and Evolutionary History of the Alpine Medicinal Plant *Gentiana lawrencei* var. *farreri* (Gentianaceae)

**DOI:** 10.3389/fgene.2018.00564

**Published:** 2018-11-26

**Authors:** Peng-Cheng Fu, Hui-Yuan Ya, Qi-Wei Liu, Hui-Min Cai, Shi-Long Chen

**Affiliations:** ^1^College of Life Science, Luoyang Normal University, Luoyang, China; ^2^College of Food and Drug, Luoyang Normal University, Luoyang, China; ^3^Key Laboratory of Adaptation and Evolution of Plateau Biota, Northwest Institute of Plateau Biology, Chinese Academy of Sciences, Xining, China; ^4^Qinghai Provincial Key Laboratory of Crop Molecular Breeding, Xining, China

**Keywords:** genetic structure, evolutionary history, refugia, *Gentiana lawrencei* var. *farreri*, Qinghai-Tibetan Plateau, microsatellites, chloroplast DNA

## Abstract

Understanding the genetic structure and evolutionary history of plants contributes to their conservation and utilization and helps to predict their response to environmental changes. The wildflower and traditional Chinese and Tibetan medicinal plant *Gentiana lawrencei* var. *farreri* is endemic to the Qinghai-Tibetan Plateau (QTP). To explore its genetic structure and evolutionary history, the genetic diversity, divergence, and demographics were analyzed in individuals from 31 locations across the QTP using 1 chloroplast marker and 10 nuclear microsatellite loci. High genetic diversity was detected in *G. lawrencei* var. *farreri*, and most of the genetic variance was found within populations. Values of *F*_ST_ in *G. lawrencei* var. *farreri* from nuclear microsatellite and chloroplast data were 0.1757 and 0.739, respectively. The data indicated the presence of isolation by distance. The southeast edge of the QTP was the main refugium for *G. lawrencei* var. *farreri*, and one microrefugium was also detected in the plateau platform of the QTP. Both nuclear microsatellite and chloroplast data indicated that the populations were divided into two geographically structured groups, a southeast group and a northwest group. The current genetic pattern was mainly formed through recolonization from the two independent refugia. Significant melt was detected at the adjacent area of the two geographically structured groups. Approximate Bayesian computation showed that the northwest group had diverged from the southeast group, which then underwent population expansion. Our results suggest that the two-refugia pattern had a significant impact on the genetic structure and evolutionary history of *G. lawrencei* var. *farreri*.

## Introduction

As the highest and largest plateau in the world, the Qinghai-Tibetan Plateau (QTP) has experienced drastic environmental and climate changes during the past several million years (Zhang et al., 2000; Zheng et al., 2002). The fragile ecosystem of the plateau is undergoing drastic changes at present due to the effects of global climate change (e.g., Che et al., 2014; Ma et al., 2017). Exploring the ways in which organisms responded to environmental and climate changes in the past may help us understand and predict how organisms will respond to such changes in the future.

The QTP is one of the world’s biodiversity hot spots, with a high density of endemic plant species (Myers et al., 2000). The QTP has thus recently received a great deal of attention from botanists. The evolutionary dynamics of a number of plant species in the QTP have been explored; these include *Saxifraga* (Ebersbach et al., 2017; Gao et al., 2017), *Rhodiola* (Li et al., 2018), and *Spiraea* (Khan et al., 2018). Additional examples have been cited in a number of recent reviews (Qiu et al., 2011; Liu et al., 2014; Wen et al., 2014; Favre et al., 2015; Sun et al., 2017; Xing and Ree, 2017; Yu et al., 2017). These studies indicated that evolutionary patterns varied among different taxa, and no common evolutionary models have emerged. However, it is undoubtedly true that geological and climatic changes in the QTP had triggered plant speciation and diversification in this area (Wen et al., 2014; Favre et al., 2016). Although more and more taxa have been studied in recent years, the QTP is still poorly studied in comparison to mountainous areas in Europe and the Americas (Favre et al., 2015; Sun et al., 2017). Therefore, more research is required in order to explore the dynamics of speciation and the evolutionary history of plants in the QTP.

As a typical alpine taxon, *Gentiana* L. occurs in numerous mountain systems of the world and encompasses 362 species (Ho and Liu, 2001). Hosting c. 250 species (Ho and Pringle, 1995), the mountain ranges surrounding the QTP are the main center of diversity for *Gentiana* and acted as the primary source area for dispersals to other parts of the world (Favre et al., 2016). The diversification within *Gentiana* has been influenced by geological and climatic changes in the QTP (Favre et al., 2016), similar to many other alpine taxa (Wen et al., 2014; Matuszak et al., 2016; Yu et al., 2017). The previous studies focused mainly on the phylogenetics and evolution in or above the section level (e.g., Yuan et al., 1996; Zhang et al., 2009; Favre et al., 2016; Sun et al., 2018b). Few studies have explored intraspecific population genetics. Population genetics was considered in just one species in the section *Cruciata* Gaudin (Lu et al., 2015), which is endemic to the QTP, and seven species in the section *Ciminalis* (Adanson) Dumorti (Diadema et al., 2005; Kropf et al., 2006; Christe et al., 2014), which is endemic to Europe. These population genetics studies examined how species in the genus diverged with the changing environments and offered insights into their mode of speciation. As a diversity center and source area, more taxa from the QTP should be studied to understand the speciation pattern and evolutionary dynamics of *Gentiana*.

In this study, we focused on *Gentiana lawrencei* var. *farreri* T. N. Ho (Gentianaceae), which belongs to section *Kudoa* (Masamune) Satake and Toyokuni ex Toyokuni and is endemic to the QTP. It lives in alpine meadows and wet meadows with altitude from 2,400 to 4,600 m. Its flower and fruit period is from August to October and pollinators are insects, mostly *Bombus*, *Thripidae*, and *Formica* (Hou et al., 2009), and seeds are not dispersed very far from the mother plant. The species is a perennial wildflower that has been domesticated for horticultural gardening (Rybczyński et al., 2015). It is also used in traditional Chinese and Tibetan medicine (Ho and Liu, 2001; Yang et al., 2012). In the QTP, *G. lawrencei* var. *farreri* is a very common species in its section *Kudoa*, in which most taxa have geographically limited distributions (Ho and Pringle, 1995). Exploring the intraspecies genetic diversification and evolutionary history of the common species can contribute to its utilization and will be helpful to understand the divergence and speciation of the section as well. Using both chloroplast DNA (cpDNA) and nuclear microsatellites, we investigated the population genetics structure and evolutionary history of *G. lawrencei* var. *farreri* from the point of matrilineal inheritance as well as nuclear inheritance.

## Materials and Methods

### Sampling

**FIGURE 1:**
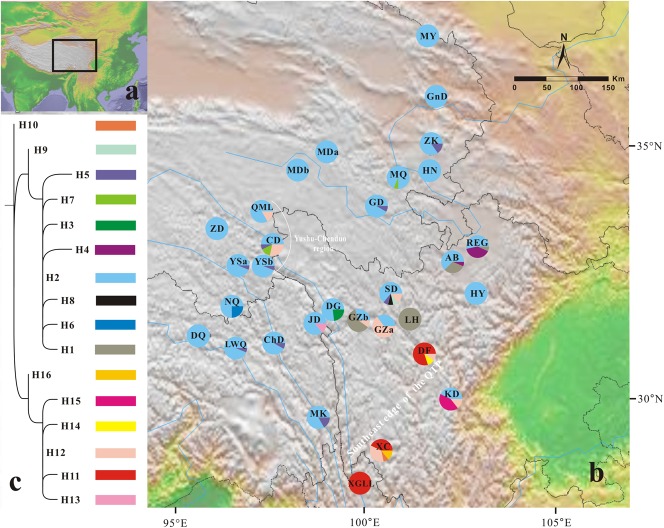
Geographical distribution of 16 chloroplast haplotypes (H1–H16) identified in *Gentiana lawrencei* var. *farreri*. **(A)** The distributions of *G. lawrencei* var. *farreri* on the Qinghai-Tibetan Plateau. **(B)** Geographical distribution of haplotypes across sampled populations. Pie charts display haplotype frequencies in each locality. **(C)** Phylogeny of the 16 chloroplast haplotypes detected in *G. lawrencei* var. *farreri*. The detailed tree with outgroups is presented in Supplementary Figure S1.

We sampled 31 populations of *G. lawrencei* var. *farreri* throughout the QTP, amounting to 423 individuals (Table 1 and Figure 1). For large populations (>100 individuals; *N*_pop_ = 28), 10–30 mature plants were randomly sampled along a single transect. Sampled individuals were at least 20 m apart. For small populations (<100 individuals; *N*_pop_ = 3), 25–50% of the plants (3–9 individuals) were sampled. Voucher specimens were deposited in the herbarium of the College of Life Science, Luoyang Normal University.

**Table 1:** Summary genetic statistics for *Gentianan lawrencei* var. *farreri*.

P.	Voucher Ref.	Locality	Latitude (N)	Longitude (E)	Altitude (m/a.s.l)	No.	Haplotype	*h*	π (10^-3^)	Na	*R*_S_	*H*_O_	*H*_E_	HW
ZK	ZH2014123	Zeku, QH	35°12′	101°26′	3938	13	H2(11), H5(2)	0.282	1.383	6.3	3.45	0.762	0.685	1
MY	Fu2015060	Mengyuan, QH	37°32′	101°16′	3500	17	H2(17)	0	0	7.6	3.46	0.818	0.706	2
REG	Fu2016022	Ruoergai, SC	33°12′	102°24′	3545	15	H1(1), H2(8), H4(6)	0.591	2.568	8.8	3.68	0.740	0.710	0
AB	Fu2016025	Ruoergai, SC	32°55′	101°48′	3500	16	H1(5), H2(10), H4(1)	0.542	1.532	7.9	3.43	0.806	0.749	0
HY	Fu2016028	Hongyuan, SC	32°14′	102°29′	3597	18	H2(18)	0	0	7.5	3.42	0.619	0.675	3
SD	Fu2016039	Seda, SC	32°17′	100°16′	3926	14	H2(9), H5(1), H8(1), H9(1), H12(2)	0.593	5.387	7.3	3.59	0.749	0.704	1
GZa	Fu2016049	Ganzi, SC	31°39′	100°10′	4043	28	H2(10), H12(18)	0.479	7.043	8.1	3.39	0.693	0.693	1
GZb	Fu2016058	Ganzi, SC	31°44′	99°33′	3937	25	H1(14), H2(8), H12(3)	0.568	3.406	10.4	3.85	0.820	0.764	2
DG	Fu2016060	Dege, SC	31°52′	99°04′	4093	13	H2(10), H3(3)	0.385	2.828	7.7	3.74	0.662	0.749	3
DF	Fu2016070	Daofu, SC	30°59′	101°08′	3548	10	H11(8), H12(1), H14(1)	0.378	2.233	6.5	3.66	0.790	0.744	2
KD	Fu2016089	Kangding, SC	30°04′	101°47′	4224	7	H2(3), H12(1), H15(3)	0.714	2.101	5.7	3.51	0.743	0.696	0
XGLL	Fu2016146	Xianggelila, YN	28°18′	99°45′	3881	18	H11(18)	0	0	6.4	3.18	0.585	0.633	2
XC	Fu2016158	Xiangcheng, SC	28°49′	100°03′	4628	14	H10(1), H11(6), H12(5), H16(2)	0.714	4.471	7.7	3.78	0.748	0.764	3
MK	Fu2016176	Mangkang, T	29°41′	98°32′	4342	12	H2(10), H5(2)	0.3038	1.485	7.0	3.57	0.775	0.715	0
MDa	Fu2017016	Maduo, QH	35°05′	98°47′	4330	12	H2(12)	0	0	4.7	2.40	0.667	0.684	1
MDb	Fu2017018	Maduo, QH	34°42′	98°05′	4270	12	H2(12)	0	0	3.9	1.94	0.476	0.450	0
QML	Fu2017021	Qumalai, QH	33°52′	97°14′	4493	12	H2(10), H12(2)	0.303	4.456	4.5	2.10	0.667	0.624	1
CD	Fu2017022	Chenduo, QH	33°12′	97°29′	4422	14	H2(7), H5(1), H7(2), H12(4)	0.692	8.349	5.8	2.31	0.738	0.690	1
YSa	Fu2017063	Yushu, QH	32°53′	96°41′	4500	16	H2(15), H5(1)	0.125	0.613	6.6	2.95	0.611	0.609	1
ZD	Fu2017039	Zhiduo, QH	33°33′	96°03′	4323	14	H2(14)	0	0	4.2	2.23	0.460	0.462	1
YSb	Fu2017045	Yushu, QH	32°49′	97°07′	3887	12	H2(11), H5(1)	0.167	0.817	5.6	2.57	0.714	0.677	0
NQ	Fu2017076	Nangqian, QH	31°58′	96°30′	4317	16	H2(12), H6(4)	0.400	1.961	7.1	3.35	0.731	0.672	1
LWQ	Fu2017082	Leiwuqi, T	31°08′	96°29′	4187	12	H2(11), H5(1)	0.167	0.817	3.9	2.37	0.501	0.456	3
DQ	Fu2017091	Dingqing, T	31°20′	95°43′	3706	12	H2(12)	0	0	5.8	2.99	0.525	0.582	2
ChD	Fu2017127	Changdu, T	31°18′	97°29’	3970	14	H2(13), H5(1)	0.143	0.700	7.8	3.37	0.690	0.705	1
JD	Fu2017148	Jiangda, T	31°38’	98°26′	4100	14	H2(12), H13(2)	0.264	3.878	8.9	3.53	0.786	0.728	0
LH	Fu2017177	Luhuo, SC	31°44′	100°43′	4022	3	H1(3)	0	0	3.4	3.20	0.815	0.763	0
GD	Fu2017264	Gande, QH	33°59′	99°56′	4045	12	H2(11), H5(1)	0.167	0.817	5.9	3.04	0.518	0.606	1
MQ	Fu2017284	Maqin, QH	34°38′	100°35′	3485	12	H2(11), H7(1)	0.167	1.225	6.4	3.46	0.790	0.708	0
HN	Fu2017293	Henan, QH	34°49′	101°15′	3630	6	H2(6)	0	0	4.4	2.79	0.650	0.703	0
GnD	Fu2017351	Guide, QH	36°19′	101°29′	3600	10	H2(10)	0	0	7.2	3.89	0.790	0.790	1
Total/mean						423		0.263	1.873	6.48	3.17	0.692	0.674	1.10

*P., population code; No., sample size; h, gene diversity; π, nucleotide diversity; Na, average number of observed alleles per locus; R_S_, allele richness; H_O_, average observed heterozygosity over loci; H_E_, average heterozygosity over loci; HW, number of loci deviating from HW, P < 0.01. Abbreviation after localities indicate provinces as follows: QH, Qinghai; SC, Sichuan; T, Tibet; YN, Yunnan.*

### Molecular Data Acquisition

Total genomic DNA was extracted from dry leaves with a Dzup plant genomic DNA extraction kit (Sangon, Shanghai, China). Through comparison analysis of chloroplast genomes (Fu et al., 2016; Sun et al., 2018b), one divergence hot spot was identified in *ycf*1 (Forward: TGCTTGTATTATTACTTTGTGG; Reverse: TTGGATTGGTATTAGTCTGGAT) and was verified in the sampled populations. The *ycf*1 was then amplified in all of the individuals of *G. lawrencei* var. *farreri*. PCRs were performed in 25-μL volumes containing approximately 20 ng of template DNA, 1× PCR Buffer, 1.5 mM MgCl_2_, 0.25 mM of each dNTP, 0.20 mM of each primer, and 1 unit of *Taq* DNA polymerase (Takara, Dalian, China). The PCR cycling profile included an initial step of 5 min at 95°C followed by 35 cycles of denaturation at 94°C for 50 s, 45 s of annealing at 53°C, 20 s at 72°C, and a final extension at 72°C for 6 min. PCR products were sequenced in the reverse direction on an ABI 3730 xl automated capillary sequencer (Applied Biosystems, Foster City, CA, United States) with BigDye v3.1 (Applied Biosystems).

All of the samples were genotyped at 10 microsatellite loci (Law4, Law5, Law24, Law32, Law37, Law41, Law45, Law57, Law71, and Law77), which were amplified and genotyped easily, as described by Sun et al. (2018a). The forward primer 5′ ends were labeled with a fluorescent dye (FAM, HEX, or ROX) [Sangon Biotech (Shanghai) Co., Ltd.]. PCR reactions and profiles followed Sun et al. (2018a). The PCR products were subsequently detected using a 3730XL Genetic Analyzer Sequencer (Applied Biosystems). Allele sizing was performed using GENEMAPPER version 4.0 (Applied Biosystems) by comparing alleles with a GeneScan-500LIZ size standard (Applied Biosystems).

### Data Analysis

#### Chloroplast Sequence Data

Sequences were aligned and edited with GENEIOUS PRO 3.5.6 (Kearse et al., 2012). Haplotypes were identified in DnaSP 5.1 (Librado and Rozas, 2009) and were deposited in GenBank (accession nos. MH481820–MH481835). Gene diversity (*h*), nucleotide diversity (π) indices, *F*_ST_ and a hierarchical AMOVA were performed in ARLEQUIN 3.5 (Excoffier and Lischer, 2010). The coefficients of differentiation *G*_ST_ were calculated using the software PERMUT (Pons and Petit, 1996) to estimate differentiation among populations. Spatial genetic structure of cpDNA haplotypes was analyzed with SAMOVA 1.0 (Dupanloup et al., 2002).

To obtain the phylogenetic relationship among haplotypes, maximum-likelihood (ML) analyses were conducted in PhyML 3.0 (Guindon and Gascuel, 2003) using the subtree pruning and regrafting (SPR) method with the HKY model which was estimated in jModelTest 2.1.7 (Darriba et al., 2012). The sequences of outgroups were extracted from the complete chloroplast genomes of *Gentiana straminea* Maxim. (Ni et al., 2016), *G. macrophy* Pall. (Wang et al., 2017) and *G. stipitata* Edgeworth (Sun et al., 2018b). The robustness of the ML trees was tested with 1000 bootstrap replicates. A statistical parsimony network was constructed and visualized utilizing PopArt 1.7 (Leigh and Bryant, 2015) to elucidate matrilineal evolutionary relationships.

The divergence times of cpDNA haplotypes were estimated using the Bayesian method implemented in BEAST 1.7.5 (Drummond et al., 2012) under the HKY substitution model, the Yule model, and an uncorrelated lognormal clock model (Drummond et al., 2006). The outgroups used in the ML analysis were used here as well. We constrained one of the nodes with a seed fossil of *G. straminea* by lognormal priors with an offset at 5.0 Ma, a mean of 0.7, and a standard deviation of 1.0, as applied by Pirie et al. (2015) and Favre et al. (2016). We ran three independent MCMC chains with 50,000,000 generations, sampling every 5,000th generation and discarding the initial 20% as a burn-in. Convergence was confirmed in TRACER 1.5^1^ and judged by ESS values (>200). Trees were summarized using TreeAnnotator 1.7.5 (Drummond et al., 2012).

To assess historical population demography, Extended Bayesian Skyline Plots (EBSP) were generated in BEAST. The Bayesian MCMC was run for 100,000,000 generations, discarding the initial 10% as a burn-in. Tajima’s *D* (Tajima, 1989) and Fu’s *F*s (Fu, 1997) were also estimated using 10,000 coalescent simulations in ARLEQUIN.

#### Microsatellite Data

The presence of scoring errors was checked using MICROCHECKER 2.2.3 (van Oosterhout et al., 2004). The raw microsatellite data was uploaded to Figshare (doi: 10.6084/m9.figshare.7195511). The null allele frequency estimates were analyzed following the Expectation Maximization algorithm (Dempster et al., 1977) in FreeNa (Chapuis and Estoup, 2007). Departure from Hardy-Weinberg equilibrium (HWE) and tests of genotypic linkage disequilibrium (LD) were carried out using Genepop 4.0 (Rousset, 2008). We calculated gene diversity according to Nei (1987) and observed heterozygosity (*H*_O_), expected heterozygosity (*H*_E_), and pairwise *F*_ST_ using ARLEQUIN 3.5 (Excoffier and Lischer, 2010). Allele richness (*R*_S_) was calculated using FSTAT 2.9 (Goudet, 2001). A hierarchical analysis of molecular variance (AMOVA; Excoffier et al., 1992) was used to further quantify genetic differentiation in ARLEQUIN with 1000 permutations. We looked for significant correlations between genetic and geographical distances using ARLEQUIN 3.5. For checking isolation by distance, *F*_ST_ was regressed against straight geographical distance and tested for significance using the Mantel test with 10,000 permutations. Recent changes of effective population sizes were tested with the Wilcoxon signed-rank test implemented in BOTTLENECK 1.2.02 (Piry et al., 1999) using the two-phase mutational model (TPM) with 10, 000 iterations.

We investigated the genetic structure among populations and individuals using the Bayesian clustering algorithm implemented in STRUCTURE 2.3.1 (Pritchard et al., 2000). We ran 10 independent replicates testing for 1–12 clusters (*K*). Each run started with a burn-in of 100,000 followed by 1 million iterations. The most likely true value of *K* was determined using the second rate of change in the likelihood distribution (*ΔK*; Evanno et al., 2005) in STRUCTURE HARVESTER 0.6.94 (Earl and vonHoldt, 2012). Graphical representation of individual cluster assignments was performed using DISTRUCT 1.1 (Rosenberg, 2004).

#### Microsatellite and Chloroplast Sequence Data

Demographic history was modeled through coalescent-based approximate Bayesian computation (ABC; for reviews see Bertorelle et al., 2010; Csillery et al., 2010). We divided populations of *G. lawrencei* var. *farreri* into two lineages identified by STRUCTURE. The first lineage (named SE) contained populations from the southeast; the second (named NW) contained populations from the northwest. In this study, we tested whether population divergence was followed by changes in effective population size. The ABC analyses were conducted using DIYABC 2.0 (Cornuet et al., 2014). First, three population divergence scenarios were tested. Scenario 1 assumed one ancestral population with an effective population size of Na that split into two populations of effective size N1 (SE) and N2 (NW) at time t1. These daughter populations were then simulated to evolve independently. Scenario 2 assumed that SE was the ancestral population and that NW diverged from SE at time t1. Scenario 3 was the same as scenario 2 but swapped SE and NW. Based on the scenario with the highest support from the population divergence data (scenario 2; see section “Results”), three scenarios with effective population size changes were then tested. Compared with scenario 2, scenario 4 assumed that NW had undergone population expansion at time t2 after diverging from SE at time t1. Scenario 5 assumed that a small number of founders (N1f) originated from SE at time t1 and that the population had undergone expansion at time t2. Scenario 6 assumed that both SE and NW had undergone population expansion. Scenarios 4, 5, 6, and 2 were compared together. In the ABC analysis, the prior distributions of all parameters were uniform, and the same range was used for common parameters between models. For all six scenarios, we assumed that microsatellites evolved under a stepwise-mutation model and that the *ycf*1 evolved under a default model, and that all individual loci had the same value. A total of 500,000 coalescent simulations were run for each scenario. The posterior probabilities of the scenarios were subsequently estimated based on (i) a direct estimate, in which the 500 data sets with summary statistics closest to the target values were extracted, and (ii) a polychotomous logistic approach that recovers the 1% closest to the simulated data sets. For the scenario with the highest support, posterior distributions for parameters were estimated using a local linear regression on the closest 1% of the simulated data sets.

## Results

### Sequence Characters and Genetic Diversity

All of the samples were amplified at the *ycf1* region. The aligned sequence of the *ycf1* region in this study was 408 bp in length. The *G. lawrencei* var. *farreri* dataset consisted of 17 base substitutions (Supplementary Table S1) that identified 16 haplotypes (H1–H16) among the 423 individuals. Within the total population of *G. lawrencei* var. *farreri*, 10 populations included a single haplotype, and 9 haplotypes were exclusive to single populations (Figure 1). The values of *h* and π ranged from 0 to 0.714 and 0 to 0.0083, respectively (Table 1). The averages of *h* and π of *G. lawrencei* var. *farreri* were 0.263 and 0.0018, respectively. Populations from the southeast generally had higher levels of genetic diversity (*h* = 0.414 and π = 0.0026) than the overall average values.

All 423 of the samples were amplified at 10 nuclear microsatellites. Among all populations, the frequencies of null allele in the 10 microsatellite loci were low (ranged from 0.002 to 0.066) thus all loci were included in following analysis. Overall, population-level indices of genetic diversity, including Na, *R*_S_, *H*_O_, and *H*_E_, were variable across populations (Table 1). The mean Na ranged from 3.4 to 10.4 with an average of 6.48. The mean *R*_S_ ranged from 1.94 to 3.89 with an average of 3.17. The mean *H*_O_ and *H*_E_ ranged from 0.460 to 0.820 and 0.450 to 0.790 and averaged 0.692 and 0.674, respectively. Populations from the southeast generally had higher levels of genetic diversity (Na = 7.3, *R*_S_ = 3.54, *H*_O_ = 0.731, and *H*_E_ = 0.720) compared to the overall average values. Among the 10 loci, the number of loci/location that deviated from HW at the population level ranged from 0 to 3 with an average of 1.10. Pairwise comparisons among the 10 microsatellite loci revealed no evidence of linkage disequilibrium (*P* > 0.05). The data from the 10 microsatellite loci used in *G. lawrencei* var. *farreri* are summarized in Supplementary Table S2.

### Phylogenetic Analysis

**FIGURE 2:**
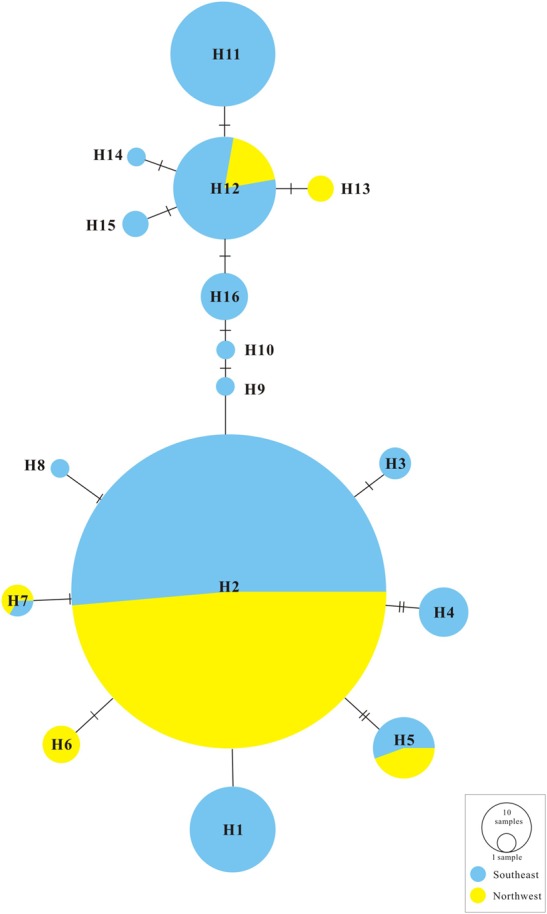
TCS statistical parsimony network of *G. lawrencei* var. *farreri* 16 chloroplast haplotypes.

**FIGURE 3:**
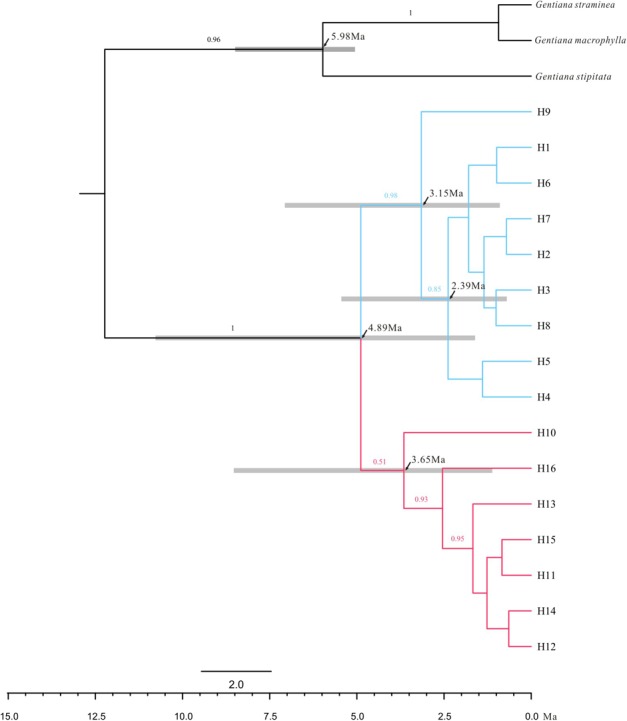
Majority rule consensus phylogenetic tree of the 16 chloroplast haplotypes detected in *G. lawrencei* var. *farreri* based on Bayesian inference. Numbers on the branches indicate Bayesian posterior probabilities. Node age estimates are marked with black arrows. Grey bars represent 95% highest posterior densities.

The ML trees of the 16 cpDNA haplotypes (Figure 1 and Supplementary Figure S1) supported two clades; this was approximately consistent with the parsimony network (Figure 2). Clade I contained 8 haplotypes (H9–H16) that were mainly distributed in the southeast. Clade II included 7 haplotypes (H1–H8); within these, H2 was a distinct high-frequency haplotype located in the center of the network. H12 was also a high-frequency haplotype located in the center of Clade I. The molecular dating analysis of the cpDNA dataset (Figure 3) estimated that the two lineages in *G. lawrencei* var. *farreri* diverged approximately 4.89 Ma (95% highest posterior density: 1.61–10.77 Ma).

### Genetic Structure

In the 16 cpDNA haplotypes detected from *G. lawrencei* var. *farreri*, H2 was dominant and appeared in 27 (87.10%) populations. All of the other haplotypes appeared in less than 10 populations. The southeast of the distribution area (populations DF, KD, XC, and XGLL) contained 8 (50%) haplotypes and YuShu-ChenDuo region (populations QML, CD and YSb) contained 4 haplotypes. SAMOVA results indicated that *F*_CT_ reached a plateau when *K* = 2 (Supplementary Table S3). Populations GZa, DF, XC, and XGLL from the south were clustered into one group, while the remaining populations formed a separate group. According to the clustering, AMOVA based on the cpDNA dataset revealed that most of the genetic variation occurred among groups (67.05%), with little further subdivision among populations within groups (6.88%) or within populations (26.07%) (Table 2). Based on cpDNA data, *F*_ST_ in *G. lawrencei* var. *farreri* was 0.739, while the value of *G*_ST_ was 0.474. The *G*_ST_ of two possible hot spots was also calculated. The values of *G*_ST_ in the southeast populations (including DF, KD, XC, and XGLL) and YuShu-ChenDuo populations (including QML, CD, and YSb) were 0.374 and 0.111, respectively.

**Table 2:** Analyses of molecular variance in *G. lawrencei* var. *farreri* based on 10 SSR loci and cpDNA. d.f., degrees of freedom.

Source of variation	d.f.	Sum of squares	Variance components	Percentage of variation
**SSR**				
Among groups	1	152.658	0.33041 Va	8.36
Among populations within groups	29	483.527	0.49988 Vb	12.65
Within populations	815	2543.453	3.1208 Vc	78.99
Total	845	3179.638	3.95109	
**cpDNA**				
Among groups	1	116.576	1.08752 Va	67.05
Among populations within groups	29	54.427	0.11164 Vb	6.88
Within populations	377	159.387	0.42278 Vc	26.07
Total	407	330.39	1.62194	

**FIGURE 4:**
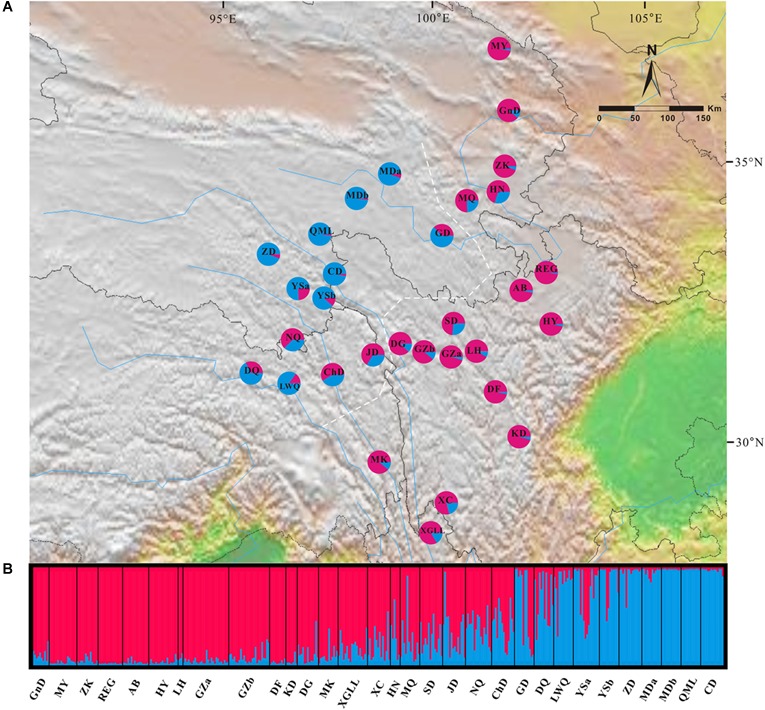
Genetic structure across the distribution range of *Gentiana lawrencei* var. *farreri*. **(A)** Geographical distribution of genetic clusters estimated by Bayesian clustering. Pie charts show the frequencies of clusters in each population; colors correspond to each cluster. **(B)** The bar plot shows the probabilities of ancestral clusters of each sample estimated by Bayesian clustering. The name of each population is shown below the bar plot. The white dash line marks the southeast group and the northwest group mentioned in the text.

In the Bayesian individual-based clustering analysis based on the microsatellite dataset, the log-likelihood values for the number of clusters (*K*) increased with each *K*-value and did not plateau (Supplementary Figure S2). The method proposed by Evanno et al. (2005) suggested that the number of clusters was 2 (*ΔK* = 2, Supplementary Figure S2). Assuming two genetic clusters, *G. lawrencei* var. *farreri* individuals sampled from the southeast were predominantly assigned to Cluster 1, while individuals from the northwest were predominantly assigned to Cluster 2 (Figure 4). AMOVA revealed that 8.36% of the genetic variance was found between groups (Va = 0.330), 12.65% among populations within species (Vb = 0.500), and 78.99% within populations (Vc = 3.121) (Table 2). The value *F*_ST_ was 0.2101, and the pairwise *F*_ST_ between the southeast edge group and the plateau platform group was 0.1533 (*P* = 0.000). The population pairwise values of *F*_ST_ are listed in Supplementary Table S4. “Isolation By Distance” showed a significant correlation between genetic and geographical distance among *G. lawrencei* var. *farreri* populations (*r*^2^ = 0.485, *P* < 0.01).

### Demographic History

Based on cpDNA data, the EBSP generated in BEAST showed that the historical population demography of *G. lawrencei* var. *farreri*, west and east groups were all constant within the most recent 200 Kya (Supplementary Figure S3). The values of Tajima’s *D* were generally negative in *G. lawrencei* var. *farreri* populations but only significant in population MQ (Supplementary Table S5). Values of Fu’s *F*_S_ were generally positive in *G. lawrencei* var. *farreri* populations, and all of the values were not significant (Supplementary Table S5). Based on microsatellite data, recent population declines were found within two (XC and MDa) of the sampled locations using the program BOTTLENECK (*P* < 0.05, Supplementary Table S6).

**FIGURE 5:**
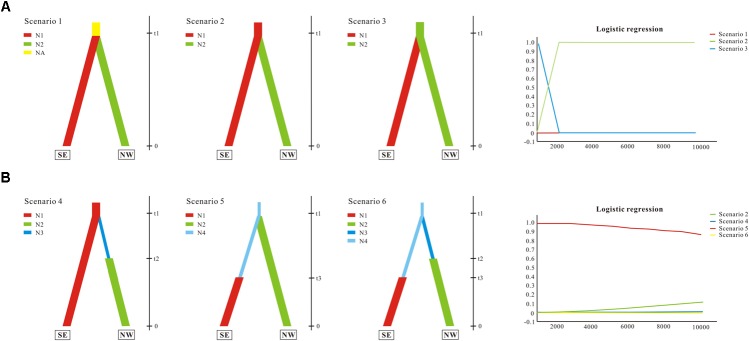
Schematic representation of models tested for *G. lawrencei* var. *farreri* population history on the Qinghai-Tibetan Plateau using approximate Bayesian computation. **(A)** Tested population divergence scenarios and their posterior probabilities. **(B)** Tested scenarios with effective population size changes and their posterior probabilities. SE represents the southeast group, and NW represents the northwest group. N (N1, N2, …) represents effective population size, and t (t1, t2, and t3) represents time. Posterior probabilities were estimated based on a polychotomous logistic approach. More details are given in Section “Materials and Methods.”

We evaluated population divergence and effective population size changes from the combined nuclear and cp data. We first compared three population divergence scenarios; the ABC simulations showed slightly higher support for scenario 2 (subpopulation NW diverged from subpopulations SE) with direct estimation (not shown); there was stronger support with logistic regression, and thus scenario 2 was favored as the most probable (Figure 5A). Effective population size changes were compared based on scenario 2. Direct estimation showed similar possibilities among different scenarios (not shown); however, logistic regression yielded strong support for scenario 5, suggesting that SE had undergone population expansion (Figure 5B). Parameter estimation based on scenario 5 showed that the mean value of N1, N2, N4, t1, and t3 were 99,300, 64,800, 82,300, 7,650, and 4,330, respectively. Although some of the estimated parameters showed low robustness, ABC analyses conclusively supported the scenario in which the northwest clade diverged from the southeast clade, which then experienced population expansion.

## Discussion

Our genetic investigation revealed that *G. lawrencei* var. *farreri* is divided into two geographically structured groups (Figures 1, 2). This genetic structure plausibly reflects the post-glacial colonization history.

### Genetic Divergence and Glacial Refugia

Due to maternal inheritance, plastid datasets can provide information on past changes in species distribution when colonization of new habitats occurs through seeds (Petit et al., 2003). Based on the cpDNA dataset, genetic diversity in the southeast edge of the QTP is significantly higher than in other regions. Since high diversity would be achieved through the mixing of already-existing genetic information but not created anew, the refugia should be identified not only by high genetic diversity but also by their genetic uniqueness (Petit et al., 2003). The southeast edge of the QTP contained half of the haplotypes, including several exclusive haplotypes, such as H10, H11, and H14-H16, which were clustered in one clade in the ML tree (Figure 1 and Supplementary Figure S1). The value of *G*_ST_ in this area was also higher than in the remaining areas. The two central haplotypes in the network (H2 and H12), which were the ancient haplotypes, occurred in this area as well. The populations situated close to the southeast edge of the QTP (JD, DG, GZb, SD, AB, and REG) were also highly divergent. Meanwhile, the diversity declined away from the southeast edge of the QTP. These characteristics are well fitted by the refugia hypothesis (Hewitt, 2000; Petit et al., 2003). Therefore, the southeast edge of the QTP appears to have been the refugium of *G. lawrencei* var. *farreri* during glaciations. The nuclear microsatellites dataset also indicated that populations from the southeast edge of the QTP had higher genetic diversity than other areas. This area is well studied and is the most important refugium for alpine plants in the QTP (reviewed in Qiu et al., 2011; Liu et al., 2014; Sun et al., 2017).

We also noted that the genetic diversity of the YuShu-ChenDuo region (populations QML, CD, and YSb) was higher than in other areas of the plateau platform. The haplotypes occurring in the plateau platform were observed from single clades in the ML tree except for H12, which occurred in both the YuShu-ChenDuo area and the southeast edge of the QTP. Although the value of *G*_ST_ in this area was much lower than in the southeast populations, it was higher than in the adjacent areas of the plateau platform. Therefore, the YuShu-ChenDuo area may be a microrefugium of *G. lawrencei* var. *farreri*. Some microrefugia had already been detected on the QTP platform in hardy plants such as *Sibiraea* (Fu et al., 2016) and *Rhodiola* sect. *Trifida* (Li et al., 2018); these have been reviewed in Qiu et al. (2011) and Liu et al. (2014). The multi-refugia pattern will have obvious impacts on the genetic structure of plant populations.

### Genetic Structure

Two evolutionary units were identified in *G. lawrencei* var. *farreri*. Based on the cpDNA data, phylogenetic relationships of haplotypes showed that the southeast and northwest areas were two geographically structured groups (Figures 1, 2). Consistent with the plastid data, the Bayesian clustering from the nuclear SSR data also indicated that populations of *G. lawrencei* var. *farreri* were divided into significantly geographical southeast and northwest groups (Figure 4), although the specific areas from the two kinds of data were not completely consistent. The inconsistence revealed by matrilineal and nuclear inheritance may be caused by pollen flow or introgression.

Mountains usually act as dispersal barriers for plants. The mountains in the distribution area of *G. lawrencei* var. *farreri*, for example, the Bayan Har Mountains and Tanggula Mountains, run northwest-southeast. If the mountains are dispersal barriers for *G. lawrencei* var. *farreri*, as, for example, the Rocky Mountains are for the barn owl (Machado et al., 2018), the plant populations would be divided into geographically northeast and southwest groups rather than the observed southeast and the northwest groups. The actual pattern could be explained by several hypotheses. One hypothesis is the two-refugia pattern of *G. lawrencei* var. *farreri*. Independent refugia during glaciation events is conducive to allopatric divergence (Petit et al., 2003). Recolonization after glaciation from independent refugia at the southeast edge of the QTP and the northwest plateau platform may have formed two genetic clades that laid the foundation for the current genetic structure. The other hypothesis is that the mountains in the QTP platform usually had low relative elevations and did not act as geographical barriers to dispersal (Yu et al., 2017). Plants that were pollinated by insects or animals and dispersed by the wind, as in *G. lawrencei* var. *farreri* (Hou et al., 2009), could spread their genetic information across the mountains at short geographical distances.

We also detected high genetic divergence and significant correlations between genetic and geographical distances in *G. lawrencei* var. *farreri*. Considering that the isolation by mountains in the plateau platform is limited for plants, we could infer that the independent refugia pattern had a significant influence on the evolutionary history of *G. lawrencei* var. *farreri*.

### Evolutionary History

Molecular dating based on cpDNA data indicated that the southeast and northwest groups diverged around 4.89 Ma when the QTP has uplift already (Favre et al., 2015). Therefore, the divergence was not caused by the uplift of the QTP, but maybe caused by environmental changes, glaciation or geographic isolation after expansion. Compared with plastid data, nuclear data reflect biparental inheritance and can yield information on evolutionary events occurring via pollen flow. The ABC analysis based on the cpDNA and nuclear data showed that the two groups were not of independent origin; the northwest group was indicated as having diverged from the southeast group. When generation time of *G. lawrencei* var. *farreri* was set to 3 years, the divergence occurred about 22,950 years ago, when the QTP was in the Last Glacial period (Zheng et al., 2002). The analysis hinted that the northwest group may be the *in situ* remnant of the last colonization from the southeast group, possibly the remnant of the last colonization after glaciation from the southeast edge of the QTP.

The ABC analysis rejected the expansion of effective population size in both groups (Scenario 6) and only favored the scenario in which southeast groups had undergone population expansion after the northwest group had diverged. However, EBSP analysis based on cpDNA indicated that the effective population sizes of the two groups and the whole species were nearly constant during the most recent 200 Kya. Recent population expansions or declines were also detected in very limited areas (MQ, and MDa and XC, respectively). The demographic histories of these populations should be explored further.

The genetic dataset indicated recolonization routes for *G. lawrencei* var. *farreri*. The main route of recolonization from the southeast edge of the QTP after the most recent glaciation was northward, along the edge of the QTP (e.g., the Hehuang Valley) to the northern end of the distribution area of *G. lawrencei* var. *farreri*. This northward route was described by Yu et al. (2017) based on species distribution models of 20 plant species in the QTP. However, recolonization from the plateau platform of the QTP tended to occur both northward and southward. The recolonization from two locations in different directions led to population mixing. Populations such as GD, MQ, HN, JD, and ChD, located at the adjacent region of the two geographically structured groups, had intermediate cluster membership and thus acted as “melting points.”

## Author Contributions

P-CF conceived and designed the experiments, collected the samples, performed the experiments, contributed reagents, materials, analysis tools, and wrote the paper. H-YY contributed reagents, materials, analysis tools, prepared figures and/or tables. Q-WL and H-MC performed the experiments and analyzed the data. S-LC analyzed the data and reviewed drafts of the paper.

## Conflict of Interest Statement

The authors declare that the research was conducted in the absence of any commercial or financial relationships that could be construed as a potential conflict of interest.

## Footnotes

1 http://tree.bio.ed.ac.uk/software/tracer/
