# Supplementary material for: Out of Refugia: Population Genetic Structure and Evolutionary History of the Alpine Medicinal Plant Gentiana lawrencei var. farreri (Gentianaceae)
Source: Front Genet. 2018 Nov 26;9:564. doi: 10.3389/fgene.2018.00564 (PMC6275180; doi:10.3389/fgene.2018.00564)
Supplement: Supplementary file 1 [file Table_1.DOCX]

Table S1 The nucleotide variation of 16 cpDNA haplotypes identified in *Gentianan lawrencei* var. *farreri*. Dots represent the first nucleotide in each column.

| Name | Nucleotide | | | | | | | | | | | | | | | | | |
| --- | --- | --- | --- | --- | --- | --- | --- | --- | --- | --- | --- | --- | --- | --- | --- | --- | --- | --- |
| H1 | G | G | A | C | T | C | T | C | A | T | G | G | C | A | C | T | C |  |
| H2 | . | . | . | . | . | . | . | . | G | . | . | . | . | . | . | . | . |  |
| H3 | . | . | . | . | . | . | . | . | G | . | . | . | . | . | . | . | T |  |
| H4 | T | . | . | . | . | G | . | . | G | . | . | . | . | . | . | . | . |  |
| H5 | . | T | C | . | . | . | . | . | G | . | . | . | . | . | . | . | . |  |
| H6 | . | . | . | . | . | . | . | . | G | G | . | . | . | . | . | . | . |  |
| H7 | . | . | . | . | . | . | . | . | G | . | . | . | . | G | . | . | . |  |
| H8 | . | . | . | . | . | . | . | T | G | . | . | . | . | . | . | . | . |  |
| H9 | . | . | . | . | . | . | . | . | G | . | . | . | . | . | . | C | . |  |
| H10 | . | . | . | . | . | . | . | . | G | . | . | . | G | . | . | C | . |  |
| H11 | . | . | . | T | G | . | . | . | G | . | . | . | G | . | T | C | . |  |
| H12 | . | . | . | T | . | . | . | . | G | . | . | . | G | . | T | C | . |  |
| H13 | . | . | . | T | . | . | . | . | G | . | . | A | G | . | T | C | . |  |
| H14 | . | . | . | T | . | . | . | . | G | . | A | . | G | . | T | C | . |  |
| H15 | . | . | . | T | . | . | A | . | G | . | . | . | G | . | T | C | . |  |
| H16 | . | . | . | T | . | . | . | . | G | . | . | . | G | . | . | C | . |  |
