# Supplementary material for: Out of Refugia: Population Genetic Structure and Evolutionary History of the Alpine Medicinal Plant Gentiana lawrencei var. farreri (Gentianaceae)
Source: Front Genet. 2018 Nov 26;9:564. doi: 10.3389/fgene.2018.00564 (PMC6275180; doi:10.3389/fgene.2018.00564)
Supplement: Supplementary file 2 [file Table_2.DOCX]

Table S2 The summarized information of 10 microsatellite loci in *Gentianan lawrencei* var. *farreri*.

| Locus | Size (bp) | Nt | Ho | He | HW |
| --- | --- | --- | --- | --- | --- |
| Law4 | 194-284 | 33 | 0.920 | 0.903 | 4 |
| Law5 | 151-280 | 29 | 0.953 | 0.900 | 7 |
| Law24 | 128-198 | 25 | 0.653 | 0.818 | 1 |
| Law32 | 209-278 | 24 | 0.737 | 0.879 | 1 |
| Law37 | 116-173 | 11 | 0.366 | 0.336 | 0 |
| Law41 | 257-397 | 27 | 0.615 | 0.861 | 2 |
| Law45 | 157-221 | 24 | 0.811 | 0.889 | 4 |
| Law57 | 210-369 | 38 | 0.574 | 0.929 | 7 |
| Law71 | 157-241 | 15 | 0.442 | 0.674 | 8 |
| Law77 | 141-216 | 16 | 0.352 | 0.359 | 0 |
| Mean |  | 24.2 | 0.642 | 0.755 | 3.4 |

Nt, total number of observed allele; Ho, average observed heterozygosity over loci; He, average heterozygosity over loci; HW, number of loci deviating from HW, P<0.01.
