# Supplementary material for: Out of Refugia: Population Genetic Structure and Evolutionary History of the Alpine Medicinal Plant Gentiana lawrencei var. farreri (Gentianaceae)
Source: Front Genet. 2018 Nov 26;9:564. doi: 10.3389/fgene.2018.00564 (PMC6275180; doi:10.3389/fgene.2018.00564)
Supplement: Supplementary file 3 [file Table_3.DOCX]

Table S3 Results from the SAMOVA based on cpDNA dataset.

| K | FST | FCT |
| --- | --- | --- |
| 2 | 0.73934 | 0.77051 |
| 3 | 0.74577 | 0.69893 |
| 4 | 0.74631 | 0.7038 |
| 5 | 0.74402 | 0.69974 |
| 6 | 0.72388 | 0.66969 |
| 7 | 0.71959 | 0.66685 |
