# Supplementary material for: Out of Refugia: Population Genetic Structure and Evolutionary History of the Alpine Medicinal Plant Gentiana lawrencei var. farreri (Gentianaceae)
Source: Front Genet. 2018 Nov 26;9:564. doi: 10.3389/fgene.2018.00564 (PMC6275180; doi:10.3389/fgene.2018.00564)
Supplement: Supplementary file 5 [file Table_5.DOCX]

Table S5 Tajima’s *D* and Fu’s *F_S_* in populations of *Gentianan lawrencei* var. *farreri*.

| P. | Tajima's D | Tajima's D p-value | FS | FS p-value |
| --- | --- | --- | --- | --- |
| ZK | -0.350 | 0.337 | 1.317 | 0.689 |
| MY | 0.000 | 1.000 | 0.000 | N.A. |
| REG | 1.850 | 0.984 | 1.029 | 0.705 |
| AB | 0.095 | 0.621 | 0.037 | 0.372 |
| HY | 0.000 | 1.000 | 0.000 | N.A. |
| SD | -0.476 | 0.351 | 0.310 | 0.585 |
| GZa | 2.228 | 0.989 | 7.102 | 0.993 |
| GZb | -0.569 | 0.297 | 2.067 | 0.874 |
| DG | 0.612 | 0.737 | 2.837 | 0.893 |
| DF | -0.507 | 0.325 | 0.300 | 0.451 |
| KD | 0.206 | 0.652 | -0.237 | 0.230 |
| XGLL | 0.000 | 1.000 | 0.000 | N.A. |
| XC | 1.483 | 0.922 | 0.960 | 0.736 |
| MK | -0.248 | 0.359 | 1.384 | 0.685 |
| MDa | 0.000 | 1.000 | 0.000 | N.A. |
| MDb | 0.000 | 1.000 | 0.000 | N.A. |
| QML | -0.324 | 0.408 | 4.028 | 0.959 |
| CD | 0.782 | 0.787 | 2.761 | 0.913 |
| YSa | -1.498 | 0.062 | 0.177 | 0.269 |
| ZD | 0.000 | 1.000 | 0.000 | N.A. |
| YSb | -1.451 | 0.053 | 0.432 | 0.372 |
| NQ | 0.838 | 0.816 | 2.177 | 0.847 |
| LWQ | -1.451 | 0.065 | 0.432 | 0.381 |
| DQ | 0.000 | 1.000 | 0.000 | N.A. |
| ChD | -1.481 | 0.055 | 0.296 | 0.317 |
| JD | -0.579 | 0.339 | 3.843 | 0.953 |
| LH | 0.000 | 1.000 | 0.000 | N.A. |
| GD | -1.451 | 0.065 | 0.432 | 0.354 |
| MQ | -1.629 | 0.019* | 1.054 | 0.590 |
| HN | 0.000 | 1.000 | 0.000 | N.A. |
| GnD | 0.000 | 1.000 | 0.000 | N.A. |
| Mean | -0.126 | 0.621 | 1.056 | N.A. |
| s.d. | 0.943 | 0.381 | 1.625 | N.A. |
