# Supplementary material for: Out of Refugia: Population Genetic Structure and Evolutionary History of the Alpine Medicinal Plant Gentiana lawrencei var. farreri (Gentianaceae)
Source: Front Genet. 2018 Nov 26;9:564. doi: 10.3389/fgene.2018.00564 (PMC6275180; doi:10.3389/fgene.2018.00564)
Supplement: Supplementary file 6 [file Table_6.DOCX]

Table S6 Wilcoxon test results with TPM model in BOTTLENECK using microsatellite dataset.

| Population | deficiency | excess |
| --- | --- | --- |
| ZK | 0.68750 | 0.34766 |
| MY | 0.24609 | 0.78418 |
| REG | 0.06543 | 0.94727 |
| AB | 0.36719 | 0.67383 |
| HY | 0.34766 | 0.68750 |
| SD | 0.21582 | 0.81250 |
| GZa | 0.13770 | 0.88379 |
| GZb | 0.31250 | 0.72168 |
| DG | 0.27832 | 0.75391 |
| DF | 0.72168 | 0.31250 |
| KD | 0.13770 | 0.88379 |
| XGLL | 0.27832 | 0.75391 |
| XC | 0.99316 | 0.00923* |
| MK | 0.21583 | 0.81500 |
| MDa | 0.97266 | 0.03906* |
| MDb | 0.14844 | 0.94531 |
| QML | 0.57813 | 0.50000 |
| CD | 0.42188 | 0.65625 |
| YSa | 0.21289 | 0.82031 |
| ZD | 0.01367 | 0.99023 |
| YSb | 0.53125 | 0.53125 |
| NQ | 0.53906 | 0.50000 |
| LWQ | 0.34766 | 0.68750 |
| DQ | 0.08008 | 0.93457 |
| ChD | 0.24805 | 0.78711 |
| JD | 0.06445 | 0.97559 |
| LH | 0.54492 | 0.05000 |
| GD | 0.00244 | 0.99854 |
| MQ | 0.53906 | 0.50000 |
| HN | 0.47266 | 0.57813 |
| GnD | 0.83887 | 0.18750 |
