# Supplementary figures and images for: Out of Refugia: Population Genetic Structure and Evolutionary History of the Alpine Medicinal Plant Gentiana lawrencei var. farreri (Gentianaceae)
Source: Front Genet. 2018 Nov 26;9:564. doi: 10.3389/fgene.2018.00564 (PMC6275180; doi:10.3389/fgene.2018.00564)

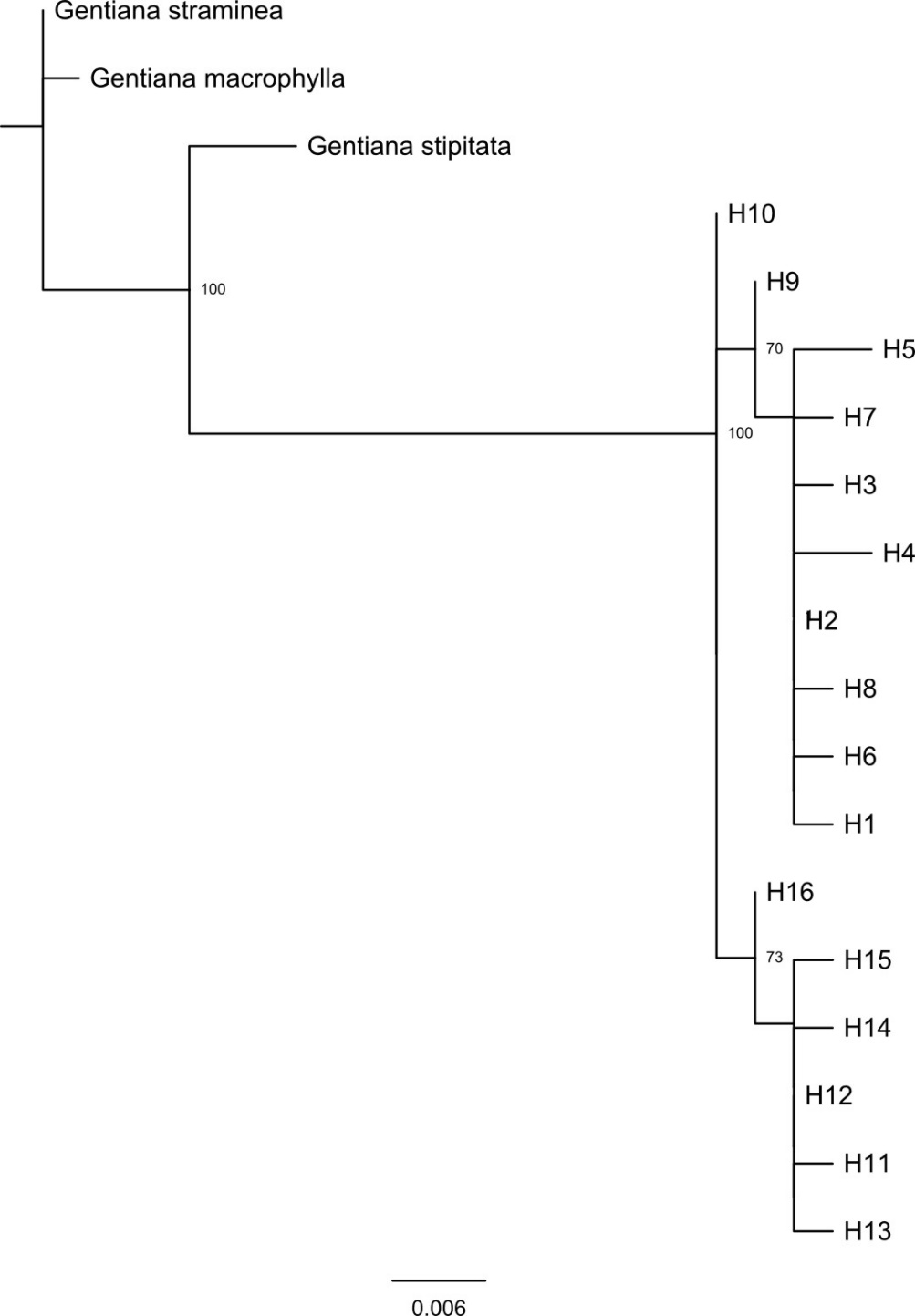


Fig. S1 The ML tree of the 16 chloroplast haplotypes detected in *G. lawrencei* var. *farreri*.

Supplement: Supplementary file 7 [file Data_Sheet_1.docx]
