# Supplementary material for: Out of Refugia: Population Genetic Structure and Evolutionary History of the Alpine Medicinal Plant Gentiana lawrencei var. farreri (Gentianaceae)
Source: Front Genet. 2018 Nov 26;9:564. doi: 10.3389/fgene.2018.00564 (PMC6275180; doi:10.3389/fgene.2018.00564)
Supplement: Supplementary file 8 [file Data_Sheet_2.docx]

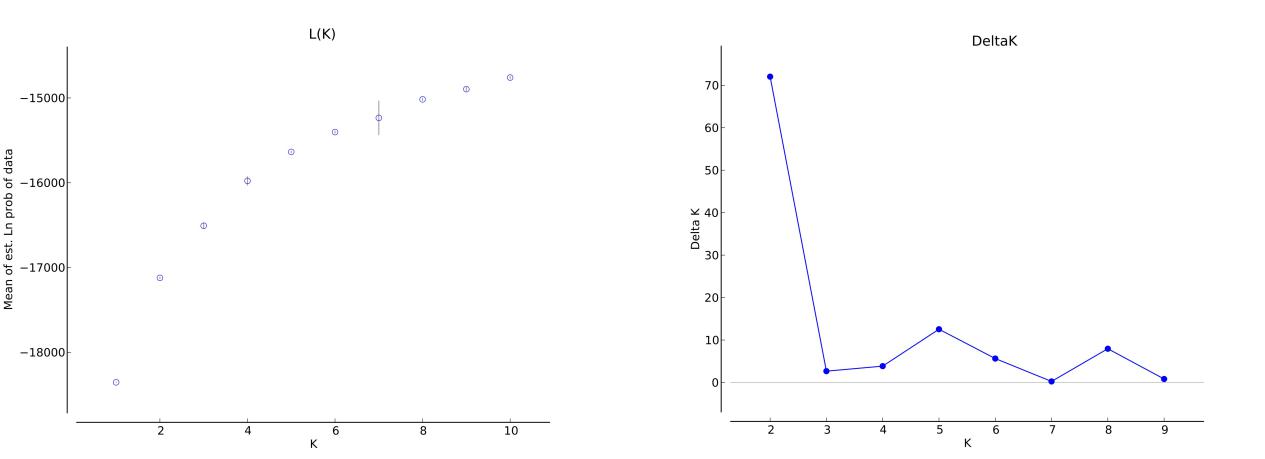


Fig. S2 Mean values for the log probability and *ΔK* (calculated according to Evanno *et al*, 2005) of the *Gentianan lawrencei* var. *farreri* microsatellite data against the assumed number of clusters (K) calculated using STRUCTURE.
