# Supplementary material for: Out of Refugia: Population Genetic Structure and Evolutionary History of the Alpine Medicinal Plant Gentiana lawrencei var. farreri (Gentianaceae)
Source: Front Genet. 2018 Nov 26;9:564. doi: 10.3389/fgene.2018.00564 (PMC6275180; doi:10.3389/fgene.2018.00564)
Supplement: Supplementary file 9 [file Data_Sheet_3.docx]

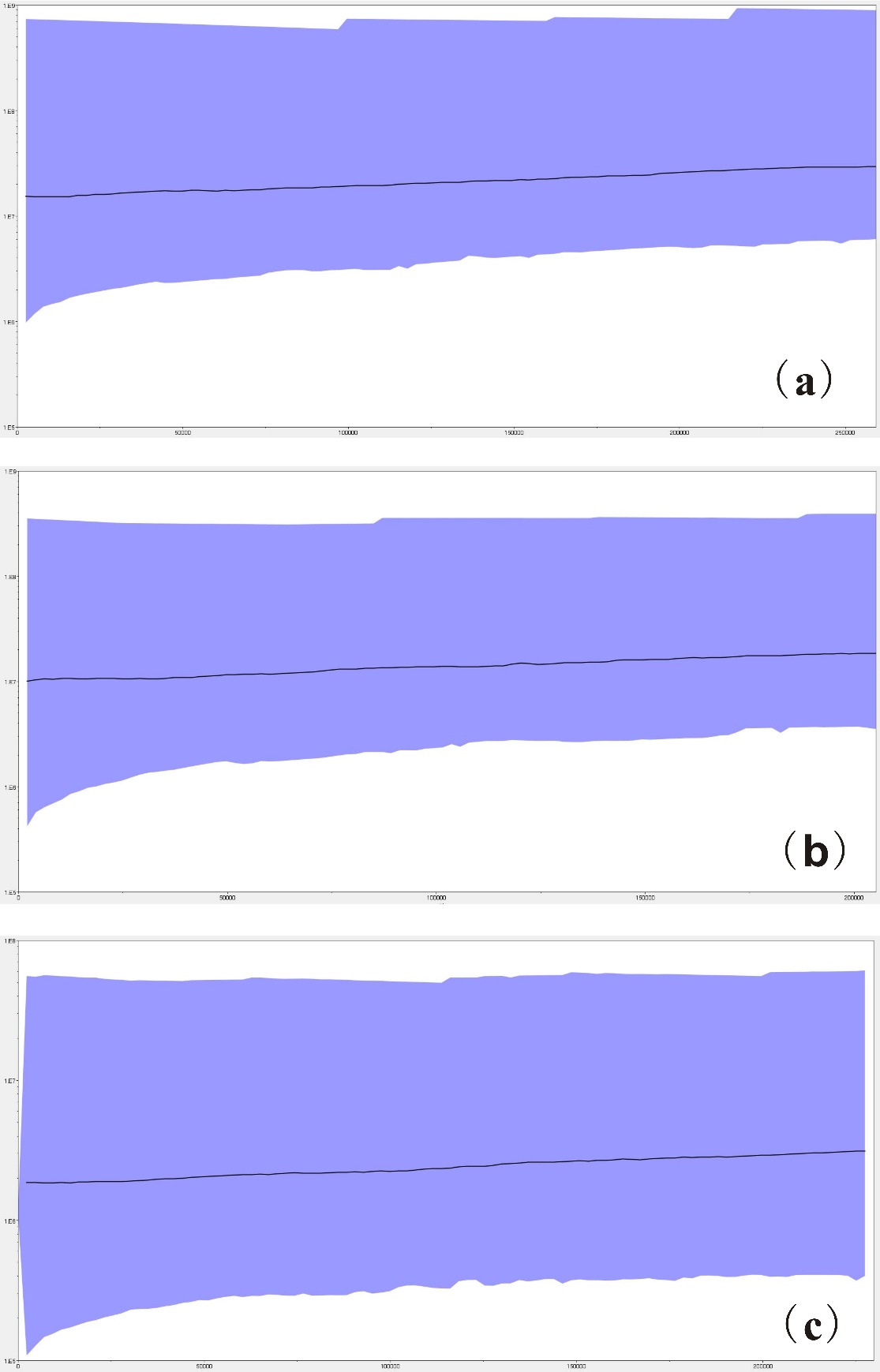


Fig. S3 Demographic histories of (a) all populations of *G. lawrencei* var. *farreri*; (b) southeast group; and (c) northwest group estimated using the Bayesian skyline plot method from chloroplast sequences. Thick black line represent the median inferred effective population size through time and fine blue lines represent 95% highest posterior density (HPD) intervals. Time is measured in years and is based on a molecular clock for chloroplast sequences.
